# A protocol for a cluster randomised feasibility study of an adolescent incentive intervention to increase uptake of HPV vaccination among girls

**DOI:** 10.1186/s40814-017-0126-y

**Published:** 2017-03-06

**Authors:** Alice S. Forster, Victoria Cornelius, Lauren Rockliffe, Laura A. V. Marlow, Helen Bedford, Jo Waller

**Affiliations:** 1 Research Department of Behavioural Science and Health, UCL, Gower Street, London, WC1E 6BT UK; 2 Imperial Clinical Trials Unit, School of Public Health, Imperial College London, Stadium House, 68 Wood Lane, London, W12 7RH UK; 3 Institute of Child Health, UCL, 30 Guilford Street, London, WC1N 1EH UK

**Keywords:** Vaccination, Reward, Adolescent, Papillomavirus Vaccines, Motivation

## Abstract

**Background:**

Uptake of the human papillomavirus (HPV) vaccine in the UK is good, but there are pockets of the community who remain unprotected. Immunisation teams usually require written parental consent for a girl to receive the vaccine. Evidence suggests that uptake of the vaccine might be improved by promoting consent form return (if returned, forms are likely to grant consent). Incentivising girls to return consent forms is a promising approach to promoting consent form return. Before testing the efficacy of an incentive intervention in a randomised controlled trial (RCT), we must first establish whether the RCT is feasible. In this randomised feasibility study, we aim to establish the feasibility of conducting a cluster RCT of an adolescent incentive intervention to increase uptake of HPV vaccination.

**Methods:**

At least six schools will be randomised to either an incentive intervention arm or a standard invitation arm. Girls in standard invitation arm schools will receive the usual HPV vaccine programme invitation materials. Girls attending schools in the incentive intervention arm will receive the standard invitation and will also be told that they will receive an incentive if they return their consent form (regardless of whether consent is granted or denied). The incentive is being entered into a prize draw to win a retail voucher. Feasibility objectives include estimating the schools’ and parents’ willingness to participate in the study and be randomised; response rates to questionnaires; the extent of missing data; the girls’ and parents’ attitudes towards the incentive offered; school staff experiences of participating, fidelity to the trial procedures, data on any unintended consequences and the possible mechanisms of action, and proof-of-concept evidence of the effect of the intervention on consent form return rates and uptake of the vaccine. Analysis of feasibility outcomes will primarily be descriptive. Consent form return rates and uptake of the vaccine will be presented by trial arm without comparison.

**Discussion:**

Incentivising HPV vaccine consent form return may promote HPV vaccine uptake. This study will provide the evidence needed to establish whether testing this incentive intervention using a RCT design in the future is feasible.

**Trial registration:**

ISRCTN72136061

## Background

Human papillomavirus (HPV) is causally related to cancers of the cervix uteri, penis, vulva, vagina, anus and oropharynx. The development and worldwide implementation of vaccines against HPV has the potential to substantially reduce the burden of HPV-related cancers. A quadrivalent HPV vaccine is used in a UK HPV vaccination programme. This vaccine provides protection against HPV types 16 and 18 that are known to cause up to 80% of HPV-related cancers (depending on cancer site) [1, 2], as well as against HPV types 6 and 11 which cause most anogenital warts. Vaccination works best if administered to HPV-naïve individuals and in younger populations [3].

While uptake of the vaccine is high in England (89% of 12–13-year olds had at least one dose of the two-dose series in 2014/2015 [4]), there are pockets of the population who remain unvaccinated and there is huge variability in uptake between areas (from 68% in Kensington and Chelsea to over 99% in the Isle of Wight). Research exploring the association between socio-demographic characteristics and uptake of HPV vaccination has suggested that girls from some Black and Asian Minority Ethnic (BAME) backgrounds are less likely to receive HPV vaccination than girls from White British backgrounds [5, 6]. This remains the case when controlling for deprivation [7]. While high uptake in the UK should offer herd protection to those who remain unvaccinated, evidence of assortivity of sexual mixing exists [8, 9] (i.e. partnerships are more likely within ethnic groups than between them). As HPV is sexually transmitted, this may mean there are population subgroups who do not benefit from herd protection, thereby compounding disease inequalities. There is very little evidence about what might work in a UK setting to increase uptake of HPV vaccination [10, 11]. If an intervention is to be rolled out nationally, it will need to be simple and easy to implement given the increasing workload of immunisation teams (the child flu vaccine and a meningitis vaccine protecting against types A, C, W and Y vaccine have recently also be added to the immunisation schedule).

Informed consent is required for girls to receive the HPV vaccine. While girls themselves may consent to vaccination if they are deemed sufficiently competent, in practice immunisation, teams usually require written consent from their legal guardian [12]. In general, consent forms are sent home from school with the girl and are returned to the school in the same way. Consent forms must be returned regardless of the parent’s decision (granting consent to vaccinate or not). Unpublished data show that around 60% of consent forms are returned without prompting and of the remaining 40%, around half will consent to vaccination if followed up by a (time consuming) telephone call from an immunisation nurse [13]. This suggests that there is a group of parents who are open to providing consent, but who may not do so without prompting. Interventions aimed at promoting return of the consent form may, therefore, increase vaccination uptake. Incentives may be a promising approach, particularly as incentives work best for one-off behaviours [14–16], with the incentive being targeted at consent form return and not vaccination receipt. Various definitions of incentives have been used previously [17, 18] and often to suit the topic of interest. In the context of the present study, incentives will be defined as financial and non-financial rewards or penalties that may have a monetary or exchange value and are contingent on the target behaviour.

### Evidence for incentives in childhood immunisation

There is varying evidence from reviews about the use of financial incentives to increase uptake of immunisation. One systematic review [19] found that incentive interventions result only in a small increase in uptake, and another systematic review and meta-analysis reported that the evidence is not strong for their use in low- and middle-income countries [20]. A further systematic review found few studies that had examined effectiveness of incentives to increase uptake of preschool immunisations and only one had reported on the cost and consequences of such an intervention [21]. Contrarily, in a descriptive review of the literature, Sutherland et al [16] concluded that there is evidence that “modest incentives” increase uptake of vaccines and Dempsey et al. [22] argue that incentive interventions to increase vaccine uptake deserve further investigation. Models of optimising vaccination uptake have also recently been published that propose using incentives in this context (e.g. [23]). However, no trials have considered incentives provided contingent on vaccination consent form return^1^ and few studies have offered incentives to the child/adolescent rather than the parents or health professional. One experimental study found hepatitis B consent form return rates in Californian (USA) schools to be higher where classes were offered an incentive contingent on all consent forms being returned with 5 days of receipt [24] and as part of an Australian service evaluation, schools were offered the opportunity to receive an incentive if >90% of vaccination consent forms were returned [25]. However the results for incentives were not reported. In one UK trial, Mantzari et al. offered incentives to older adolescent girls (aged 17–18) to receive the HPV vaccine as part of a catch-up programme (delivered through GPs and pharmacies) [26]. The tested incentive comprised a total of £45 worth of Love2Shop retail vouchers, which are redeemable at over 20,000 UK shops: £20 for receiving doses one and three and £5 for receiving the second dose. The incentive increased uptake of the first and third doses. The offer of incentives did not affect the quality of the girls’ decision to get the vaccine, as measured by the congruence between their attitudes towards the vaccine and their vaccination status. A review of methods to increase postal questionnaire response rates found a two-fold improved response rate if monetary incentives were offered [27], although this was not necessarily among adolescents in a school-based setting.

### Mechanisms of action

Incentives in this context may work because they increase the perceived value of returning the consent form (congruent with expectancy value and economic models [28–33]). Incentives might also result in individuals allocating cognitive capacity to efforts to receive the incentive [34] or the immediate reward may enhance the short-term benefits of returning the consent form [35]. Incentives may also function as a way of focusing an individual’s attention to something that they had previously not considered [36, 37]. Among adolescents, there may also be a role of fear of missing out on something one’s peers are experiencing [38] and some evidence from neuroscience suggests that reward-related brain activation is linked to improved memory, implying that incentives make a behaviour more likely because the individual remembers it [39].

### Concerns about incentives

Use of incentives to improve health-related behaviours is generally acceptable to many stakeholders [40, 41], but varies depending on incentive type, its effectiveness, and the nature of the health behaviour being targeted [42]. Sociodemographic variation in attitudes to incentives has also been observed [17, 42], including ethnic variations, which are likely due to the acceptability of the behaviour the incentive is targeting, rather than the incentive itself [17]. Cash incentives are seen as less acceptable [41], and there is concern about the potential for abuse and fear of being part of a “Nanny State”. There is also concern about incentives undermining parental choice in immunisation decisions [26, 43]; alternatively, girls “pestering” their parents may encourage parents to engage with the decision.

We have conducted formative research with teachers and girls, as well as consulting our study user groups comprising parents and immunisation nurses, to choose an incentive that is acceptable to all vaccine stakeholders, as well as likely to be sufficiently motivational to girls. It will also be important to consider acceptability on a larger scale, as well as monitoring unintended consequences.

### The present study

It will be necessary to test the efficacy of the incentive intervention in increasing HPV vaccination uptake using a randomised controlled trial (RCT) design. Before this is appropriate, we must establish whether the RCT is feasible and establish parameters needed to design the RCT. The proposed study is a randomised feasibility study. Findings will inform the decision to conduct a future RCT and will provide proof-of-concept evidence.

It is not our intention that incentives should be offered to all girls as standard as part of the vaccination programme (should incentives be found effective at improving uptake), but that they be a tool that immunisation providers may use to improve uptake in schools with particularly low coverage.

### Aim

The aim of this study is to establish the feasibility of conducting a cluster RCT of an adolescent incentive intervention to increase uptake of HPV vaccination among girls.

### Objectives

1. Gain an estimate of school participation rates, parent participation rates, response rates to a parental questionnaire and response rates to girls’ questionnaire.
2. Assess data quality and completeness.
3. Determine the acceptability of the intervention and acceptability and fidelity of the trial procedures.
4. Estimate the cost per returned consent form and cost per consent form that is agreeing to vaccination.
5. Identify longer term cost components that need to be considered in a future economic evaluation.
6. Obtain data on unintended consequences of the intervention and mechanisms of action.
7. Generate proof-of-concept evidence of the effect of the intervention on HPV vaccination uptake.
8. Generate proof-of-concept evidence of the effect of the intervention on consent form return rates.

## Methods/design

### Trial design

**Fig. 1:**
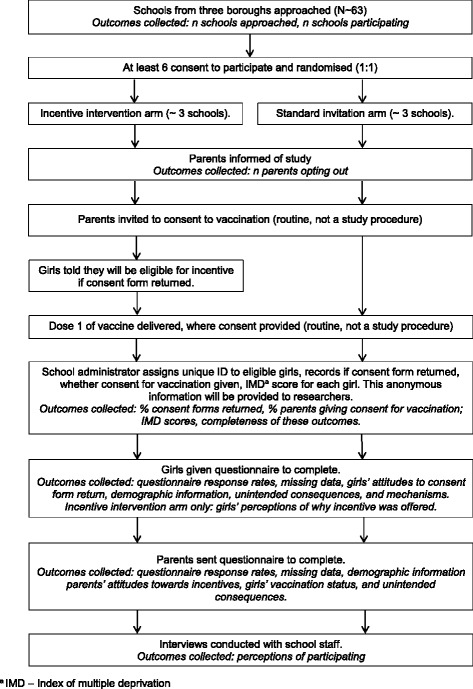
Trial flow diagram

The trial design is a two-arm cluster randomised feasibility trial, with equal allocation to each arm (see Fig. 1).

### Setting

The study will be conducted in the London boroughs of Enfield, Lambeth and Southwark. In Enfield, uptake of dose one of the two-dose HPV vaccine schedule is 75% (the fourth lowest in the country; [4]) and around 49% of residents are from a non-White British ethnic background (predominantly other White^2^ and Black or Black British Caribbean; [44]). Uptake of dose one in Lambeth is 82% (average for London, but below average for England; [4]) and 60% of residents are from a non-White British ethnic background, mainly Black African and Black Caribbean [45]. Southwark has a similar dose one uptake rate to Lambeth (83%; [4]) and around 42% of residents are from a non-White background, again, mainly Black African or Black Caribbean [46].

### Eligibility criteria

Secondary schools participating in the HPV immunisation programme in Enfield, Lambeth or Southwark are eligible for the study. Within participating schools, adolescent girls in school year eight are eligible to take part. Feasibility data will also be collected from one parent of each participating girl, school staff working in participating schools and HPV-related cancer prevention stakeholders. There are no exclusion criteria.

### Interventions

The feasibility trial has two arms: (1) standard invitation and (2) incentive intervention plus standard invitation. The standard invitation arm comprises adolescents being provided with an information leaflet about the HPV vaccine and a consent form by their school, which they are asked to give to their parents (delivered by hand) and return to the school before a prescribed date. Immunisation nurses may engage in additional promotional activities with schools, and this information will be collected. Participants in the incentive intervention arm will receive the standard invitation. They will also be advised by their school form tutor and in a letter that they will be entered into a prize draw to win a £50 “Love2Shop” voucher if they return their consent form, with a 1 in 10 chances of winning. It will be made clear that they will be eligible for the incentive regardless of whether consent is given. Schools in the intervention arm will be asked to record the day the students are informed about the incentive and the name of the teacher who did this.

The incentive aims to improve uptake among the 20% of the eligible population who will consent to vaccination if prompted to return their consent form; however, for practical purposes, all girls in the intervention arm will be eligible to receive the incentive if their consent form is returned.

### Outcomes

Feasibility outcomes collected prior to interventions being administered include the schools’ and parents’ willingness to participate in the study and the schools’ willingness to be randomised (number of schools contacted, number of those who express initial interest, and number of those who participate). Feasibility outcomes collected following interventions being administered include response rates to questionnaires by the parents and girls and data completeness regarding the proportion of missing data, girls’ and parents’ socio-demographic characteristics (including ethnicity, ascertained through brief questionnaires, and index of multiple deprivation (IMD) score, ascertained using the girls’ postcodes provided by the schools). We will also explore the girls’ and parents’ attitudes towards the incentive offered (assessed through brief questionnaires, measure developed for this study) and school staff experiences of participating (ascertained through brief interviews). Data on unintended consequences and mechanisms of effect will be collected via questionnaires. Unintended consequences include the girls’ perceptions of why an incentive was offered, the girls’ attitudes towards returning future consent forms (measures developed for this study) and whether parents made an informed decision about vaccination (using questions described in [26]). Mechanisms of effect include whether the incentive increased motivation for returning the consent form, improved memory for returning the form, increased salience for returning the form, increased the short-term benefits of returning the form, increased the perceived value of returning the form and whether there is a role for girls fearing missing out on the incentive (using [38] and measures developed for this study). Trial procedures will be documented by speaking to the school staff about their fidelity to the intervention (ascertained through brief interviews) and taking a detailed description of immunisation processes performed in each school (i.e. what additions to the standard invitation were performed) by the immunisation teams and school staff (ascertained through brief interviews with school staff and email/telephone conversations with immunisation teams throughout the trial). Cost of the incentive intervention will be assessed by measuring (1) the cost per returned consent form and (2) the cost per form consenting to vaccination.

Proof of concept outcomes collected following the first vaccination dose, including initiation of the vaccination series, will be assessed by collecting data on whether consent was given to vaccination reported by schools and self-reported by parents in a brief questionnaire (measure developed for this study). Consent form return rates will be based on information collected by schools.

### Study procedures

All secondary schools in Enfield, Lambeth and Southwark will be approached. Legal guardians (usually parents) from participating schools will be written to/emailed explaining the study and those not wishing to participate were asked to opt out to a school contact.

Schools randomised to the incentive intervention arm will be sent the intervention letter to give to students and teachers informed to tell students that they will be eligible for the incentive if their consent form is returned. At the same time, parents from schools in both arms will be asked by the immunisation team to consent to their daughter having the HPV vaccine as part of the routine programme, via an invitation letter, information leaflet and accompanying consent form. These standard immunisation programme materials are distributed to the girls by form tutors and their distribution/content is unaffected by the present study.

Following dose one of the vaccine being administered to those girls with consent, the research team will give the school administrative staff a list of unique anonymised identifiers to assign to each participating girl. The administrative team will add this list to an existing dataset containing all vaccine-eligible girls and the following information: an IMD score for each girl (derived from the girls’ postcodes), whether she returned a consent form and whether consent to vaccination was given on the consent form. Administrative staff will remove the girls’ names from the dataset and provide this to the researchers to take away from the school site.

Girls will be given a questionnaire to complete in the week after vaccination day by their form tutor, as well as a questionnaire pack to hand deliver to their parents. The parents’ packs will include a FREEPOST return envelope to send the questionnaires directly to researchers. Both questionnaires will be labelled with each girl’s unique identifier. Parents with more than one daughter in year eight will be asked to think of one child and respond about that child when completing the questionnaire. Girls will complete the questionnaire during a tutor group session. School staff will be invited to participate in a brief interview in an email sent from the research team via a member of the school administrative staff.

All interviews will be conducted shortly after vaccination day, either in person or on the phone by a member of the research team. Written/audio-recorded verbal informed consent will be taken by a member of the research team. They will be audio-recorded and transcribed verbatim.

### Sample size

This is a feasibility study and one of the outcomes will be to assess the willingness of the schools to take part in the study and to be randomised. We anticipate that at least six schools will agree to participate in the study based on our pilot work. On average, in participating boroughs, there are approximately 100 girls in year eight per school. With 600 girls, we will be able to estimate the binary feasibility outcomes (participation rates, questionnaire response rates and acceptability of the trial methods) with an unadjusted precision of the 95% confidence intervals to at least ±5 percentage points assuming the most statistically conservative scenario when the proportion is 0.5.

### Allocation

Blocked randomisation will be performed by the statistical advisor using computer generated random numbers. The statistical advisor will place pre-generated randomisation lists on a list of participating schools (with names concealed) and will send school allocation to the study research assistant. The research assistant will inform participating schools of their group assignment.

### Blinding

Once allocated (and information materials sent to schools allocated to the incentive intervention arm), group allocation will be kept separately from the main dataset. Trial arms will be labelled “A” and “B” in the dataset by the statistical advisor, who will keep the key and the main analyst will be blind to the group allocation until analysis is complete. Participants will not be blind to the group allocation.

### Data management

Data will be input by a research assistant, with 10% of data entered from questionnaires checked. If accuracy is <100%, a further 10% of data entered will be checked, with the process repeated until no errors are identified. Errors will be corrected. No identifiable data will leave school sites. Interview transcripts will be fully anonymised.

### Statistical methods

As this is a feasibility study our primary focus will be on assessing the outcomes regarding trial procedures, data quality and estimates of design parameters. The analysis for the feasibility outcomes will be primarily descriptive. School characteristics will be presented by arm. Data are available on participating and non-participating schools regarding school size, whether schools are single sex/mixed, and HPV vaccine uptake in the previous year. Participating and non-participating schools will be compared descriptively based on these factors to examine how representative participating schools are of their respective boroughs. Participant characteristics at baseline will be presented overall, by arm and by school. We will estimate and report on the proportion of schools approached that participate, the proportion of parents approached who opt out of the study, the proportion of parents sent a questionnaire who return the questionnaire and the proportion of girls given a questionnaire who complete the questionnaire.

We will estimate and report on the proportion of missing data for consent form return rates, vaccination consent, girls’ IMD score and all questionnaire items.

Acceptability of the incentive among girls and parents will be assessed descriptively. Interviews will be analysed descriptively to assess the range of opinions.

Parents’ mean informed decision-making scores will be presented by cluster and by trial arm, as will the data for other unintended consequences and mechanisms of action. We will summarise by cluster and by trial arm, the proportion of girls whose parents consent to the vaccine and the proportion of consent forms returned.

The trial will be reported according to the CONSORT extension to cluster randomised trials, CONSORT extension to randomised pilot and feasibility trials and this protocol has been prepared using the SPIRIT guideline [47–49].

### Missing data

The proportion of missing data for each variable collected via questionnaire will be reported. In line with Carpenter and Kenward [50], during a blind review of the data, the lead analyst and the statistical advisor will meet to decide upon the most appropriate missing data model and will apply this prior to analysis.

### Economic evaluation

Cost per returned consent form and cost per consent form agreeing to vaccination will be defined as the total unit cost of all consumables and incentive where relevant, divided by the number of consent forms returned or the number of consent forms returned that are agreeing to vaccination (following the method of Woodhall et al. [51]).

Interviews will be conducted with HPV-related cancer prevention stakeholders to identify the long-term cost implications of the intervention, including the administration of the incentive. This preparatory work will inform the economic analysis conducted as part of the future RCT.

### Monitoring

A trial management group comprising the study team will meet at the beginning and end of the study and throughout as needed. A data monitoring committee was not deemed necessary for this feasibility study. There will be no interim analyses or stopping guidelines. All members of the study team will have access to the final dataset. We do not anticipate any adverse events, but unintended consequences of the intervention are being measured. Immunisation teams and school staff will have a contact within the research team to report any adverse events.

### Deciding to proceed to a full trial

The decision to proceed to a definitive trial will be a multifaceted one, based on the trial procedures being acceptable to participants/stakeholders and feasible to deliver and scale up.

### Ethics and dissemination

Ethical approval has been received from the UCL Research Ethics Committee (6615/002). Modifications to this protocol will be communicated to relevant parties by the principal investigator. Results will be published in a peer review journal and presented at conferences.

## Discussion

We have presented a protocol for a cluster randomised feasibility study of an adolescent incentive intervention to increase uptake of HPV vaccination among girls. The study will help provide the answers to questions required to design any main trial and whether it is appropriate to proceed with a future trial.

We have previously achieved high response rates to questionnaires completed by students in schools; however, response rates to questionnaires posted to parents are likely to be lower. Findings from this study will determine whether this approach to data collection from parents is likely to be successful in any main trial. We will also be able to observe if certain questionnaire items are often missing, suggesting that participants either did not understand them or did not find them relevant, allowing us to modify the questionnaires prior to any main trial. Interviews with school staff may also help to explain any missing data. Finally, we presently do not know if we will obtain more complete data on vaccination status by asking schools to record this data or by asking parents to report their daughter’s vaccination status. Findings of this feasibility study will suggest how best to collect data on vaccination status in any main trial.

The sample size is based on a conservative scenario and if achieved will allow us to estimate binary feasibility outcomes. For any main trial, a larger sample size will likely be required; this will be determined following this feasibility study. It is possible that schools participating in the feasibility study will be the most engaged in research. Further efforts may be required to recruit additional schools for any main trial and may require input from senior leadership to facilitate recruitment, such as local authorities. We have engaged with local authorities in the set-up of this feasibility study, so their input should be possible in any main trial.

Uptake of the HPV vaccine among eligible 12–13-year-old girls is sub-optimal in some populations. An incentive intervention to promote consent form return may result in improved uptake. This cluster randomised feasibility study will provide the evidence needed to decide if a future RCT testing the efficacy of such an intervention is feasible.

### Trial status

Recruitment started on 1 July 2016 and ended on 15 December 2016.

## Abbreviations

BAME: Black and Asian Minority Ethnic
CI: Confidence interval
HPV: Human papillomavirus
IMD: Index of Multiple Deprivation
NHS: National Health Service
RCT: Randomised controlled trial
UCL: University College London

## Footnotes

1 One trial protocol planned to offer the use of financial incentives to increase vaccination consent form return rates as part of a multi-component intervention (Skinner et al. 2015).

2 White ethnicity, but not White British, White Irish or White Gypsy or Irish traveller.

## References

[1] Forman D, de Martel C, Lacey CJ, Soerjomataram I, Lortet-Tieulent J, Bruni L, Vignat J, Ferlay J, Bray F, Plummer M, Franceschi S (2012). Global burden of human papillomavirus and related diseases. Vaccine.

[2] HPV and Cancer [http://www.cancer.gov/about-cancer/causes-prevention/risk/infectious-agents/hpv-fact-sheet]. Accessed 18 Jan 2017.

[3] Block SL, Nolan T, Sattler C, Barr E, Giacoletti KE, Marchant CD, Castellsague X, Rusche SA, Lukac S, Bryan JT (2006). Comparison of the immunogenicity and reactogenicity of a prophylactic quadrivalent human papillomavirus (types 6, 11, 16, and 18) L1 virus-like particle vaccine in male and female adolescents and young adult women. Pediatrics.

[4] Annual HPV vaccine coverage in England : 2014-15 [https://www.gov.uk/government/statistics/annual-hpv-vaccine-coverage-2014-to-2015-by-local-authority-and-area-team]. Accessed 18 Jan 2017.

[5] Fisher H, Trotter CL, Audrey S, Macdonald-Wallis K, Hickman M (2013). Inequalities in the uptake of human papillomavirus vaccination: a systematic review and meta-analysis. Int J Epidemiol.

[6] Bowyer HL, Dodd RH, Marlow LAV, Waller J (2014). Association between human papillomavirus vaccine status and other cervical cancer risk factors. Vaccine.

[7] Fisher H, Audrey S, Mytton JA, Hickman M, Trotter C (2013). Examining inequalities in the uptake of the school-based HPV vaccination programme in England: a retrospective cohort study. J Public Health (Oxf).

[8] Laumann EO, Youm Y (1999). Racial/ethnic group differences in the prevalence of sexually transmitted diseases in the United States: a network explanation. Sex Transm Dis.

[9] Turner KM, Garnett GP, Ghani AC, Sterne JA, Low N (2004). Investigating ethnic inequalities in the incidence of sexually transmitted infections: mathematical modelling study. Sex Transm Infect.

[10] Fu LY, Bonhomme LA, Cooper SC, Joseph JG, Zimet GD (2014). Educational interventions to increase HPV vaccination acceptance: a systematic review. Vaccine.

[11] Walling EB, Benzoni N, Dornfeld J, Bhandari R, Sisk BA, Garbutt J, Colditz G. Interventions to improve HPV vaccine uptake: a systematic review. Pediatrics. 2016;138:e20153863. 10.1542/peds.2015-3863 27296865

[12] Consent: the green book, chapter 2 [https://www.gov.uk/government/uploads/system/uploads/attachment_data/file/144250/Green-Book-Chapter-2-Consent-PDF-77K.pdf]. Accessed 18 Jan 2017.

[13] Tackling gaps and inequalities in girls’ uptake of the current HPV vaccination programme [http://emhf.org/wp-content/uploads/2015/10/EMHF-HPV-Symposium-April-2015-REPORT.final_.pdf]. Accessed 18 Jan 2017.

[14] Achat H, McIntyre P, Burgess M (1999). Health care incentives in immunisation. Aust N Z J Public Health.

[15] Seal KH, Kral AH, Lorvick J, McNees A, Gee L, Edlin BR (2003). A randomized controlled trial of monetary incentives vs. outreach to enhance adherence to the hepatitis B vaccine series among injection drug users. Drug Alcohol Depend.

[16] Sutherland K, Christianson JB, Leatherman S (2008). Impact of targeted financial incentives on personal health behavior: a review of the literature. Med Care Res Rev.

[17] Morgan H, Hoddinott P, Thomson G, Crossland N, Farrar S, Yi D, Hislop J, Moran VH, MacLennan G, Dombrowski SU (2015). Benefits of incentives for breastfeeding and smoking cessation in pregnancy (BIBS): a mixed-methods study to inform trial design. Health Technol Assess.

[18] Adams J, Bateman B, Becker F, Cresswell T, Flynn D, McNaughton R, Oluboyede Y, Robalino S, Ternent L, Gardner Sood B, et al: Effectiveness and acceptability of parental financial incentives and quasi-mandatory schemes for increasing uptake of vaccinations in preschool children: systematic review, qualitative study and discrete choice experiment. Health Technol Assess. 2015;19:1–176. 10.3310/hta19940 PMC4781323 26562004

[19] Jarrett C, Wilson R, O’Leary M, Eckersberger E, Larson HJ (2015). Strategies for addressing vaccine hesitancy—a systematic review. Vaccine.

[20] Bassani DG, Arora P, Wazny K, Gaffey MF, Lenters L, Bhutta ZA (2013). Financial incentives and coverage of child health interventions: a systematic review and meta-analysis. BMC Public Health.

[21] Wigham S, Ternent L, Bryant A, Robalino S, Sniehotta FF, Adams J (2014). Parental financial incentives for increasing preschool vaccination uptake: systematic review. Pediatrics.

[22] Dempsey AF, Zimet GD (2015). Interventions to improve adolescent vaccination: what may work and what still needs to be tested. Vaccine.

[23] Betsch C, Böhm R, Chapman GB (2015). Using behavioral insights to increase vaccination policy effectiveness. Policy Insights Behav Brain Sci.

[24] Unti LM, Coyle KK, Woodruff BA, Boyer-Chuanroong L (1997). Incentives and motivators in school-based hepatitis B vaccination programs. J Sch Health.

[25] Mak DB, Bulsara M, Goggin LS, Effler PV (2011). Resending a consent form and information package to non-responders increases school-based consent return rate. Aust N Z J Public Health.

[26] Mantzari E, Vogt F, Marteau TM (2015). Financial incentives for increasing uptake of HPV vaccinations: a randomized controlled trial. Health Psychol.

[27] Edwards P, Roberts I, Clarke M, DiGuiseppi C, Pratap S, Wentz R, Kwan I, Cooper R: Methods to increase response rates to postal questionnaires. Cochrane Database Syst Rev. 2007; (2):MR000008. 10.1002/14651858.MR000008.pub3 17443629

[28] Rosenstock IM, Strecher VJ, Becker MH (1988). Social learning theory and the health belief model. Health Educ Q.

[29] Camerer CF, Hogarth RM (1999). The effects of financial incentives in experiments: a review and capital-labor-production framework. J Risk Uncertain.

[30] Hertwig R, Ortmann A (2001). Experimental practices in economics: a methodological challenge for psychologists?. Behav Brain Sci.

[31] Dawes RM, Budescu DV, Erev I, Zwick R (1999). Experimental demand, clear incentives, both or neither. Games and human behavior: Essays in honor of annon rapoort.

[32] Lopes LL (1994). Psychology and economics: perspectives on risk, cooperation, and the marketplace. Ann Rev Psychol.

[33] Zwick R, Erev I, Budescu DV, Budescu DV, Erev I, Zwick R (1999). The psychological and economical perspectives on human decisions in social and interative contexts. Games and human behavior: essays in honor of annon rapoport.

[34] Marteau TM, Churchill CN (2010). Changing behaviour to improve population health. Health innovations: more for less in healthcare.

[35] Adams JE, White M, Babones S (2009). The role of time preference and perspective in socio-economic inequalities in health related behaviours. Social inequality and public health.

[36] Kane RL, Johnson PE, Town RJ, Butler M. Economic incentives for preventive care. Rockville: Agency for Healthcare Research and Quality (US); 2004.

[37] Hickey C, Chelazzi L, Theeuwes J (2011). Reward has a residual impact on target selection in visual search, but not on the suppression of distractors. Vis Cogn.

[38] Przybylski AK, Murayama K, DeHaan CR, Gladwell V (2013). Motivational, emotional, and behavioral correlates of fear of missing out. Comput Hum Behav.

[39] Kang MJ, Hsu M, Krajbich IM, Loewenstein G, McClure SM, Wang JT, Camerer CF (2009). The wick in the candle of learning: epistemic curiosity activates reward circuitry and enhances memory. Psychol Sci.

[40] Marshall HS, Proeve C, Collins J, Tooher R, O’Keefe M, Burgess T, Skinner SR, Watson M, Ashmeade H, Braunack-Mayer A (2014). Eliciting youth and adult recommendations through citizens’ juries to improve school based adolescent immunisation programs. Vaccine.

[41] NICE (2010). The use of incentives to improve health.

[42] Promberger M, Dolan P, Marteau TM (2012). “Pay them if it works”: discrete choice experiments on the acceptability of financial incentives to change health related behaviour. Soc Sci Med.

[43] Gardner B, McAteer J, Davies A, Michie S (2010). Views towards compulsory MMR vaccination in the UK. Arch Dis Child.

[44] Ethnicity and enfield [http://www.enfield.gov.uk/healthandwellbeing/info/13/enfield_people/147/ethnicity_and_language]. Accessed 18 Jan 2017.

[45] State of the Borough 2014 [http://www.lambeth.gov.uk/sites/default/files/ec-lambeth-council-state-of-the-borough-2014_0.pdf]. Accessed 18 Jan 2017.

[46] Ethnicity [http://www.lewishamjsna.org.uk/a-profile-of-lewisham/social-and-environmental-context/ethnicity]. Accessed 18 Jan 2017.

[47] Chan AW, Tetzlaff JM, Altman DG, Laupacis A, Gotzsche PC, Krleza-Jeric K, Hrobjartsson A, Mann H, Dickersin K, Berlin JA (2013). SPIRIT 2013 statement: defining standard protocol items for clinical trials. Ann Intern Med.

[48] Campbell MK, Piaggio G, Elbourne DR, Altman DG (2012). Consort 2010 statement: extension to cluster randomised trials. BMJ.

[49] Eldridge SM, Chan CL, Campbell MJ, Bond CM, Hopewell S, Thabane L, Lancaster GA, Grp PC. CONSORT 2010 statement: extension to randomised pilot and feasibility trials. BMJ. 2016;355:64. 10.1136/bmj.i5239 PMC5076380 27777223

[50] Missing data in randomised controlled trials - a practical guide [http://missingdata.lshtm.ac.uk/downloads/rm04_jh17_mk.pdf]. Accessed 18 Jan 2017.

[51] Woodhall SC, Nichols T, Alexander S, da Silva FC, Mercer CH, Ison C, Gill ON, Soldan K (2015). Can we use postal surveys with anonymous testing to monitor chlamydia prevalence in young women in England? Pilot study incorporating randomised controlled trial of recruitment methods. Sex Transm Infect.

